# 

**DOI:** 10.1192/bjb.2025.10188

**Published:** 2026-08

**Authors:** Jonathan M. Hurlow

**Affiliations:** Secure Services, Birmingham and Solihull Mental Health NHS Foundation Trust, Birmingham, UK.

**Figure:**
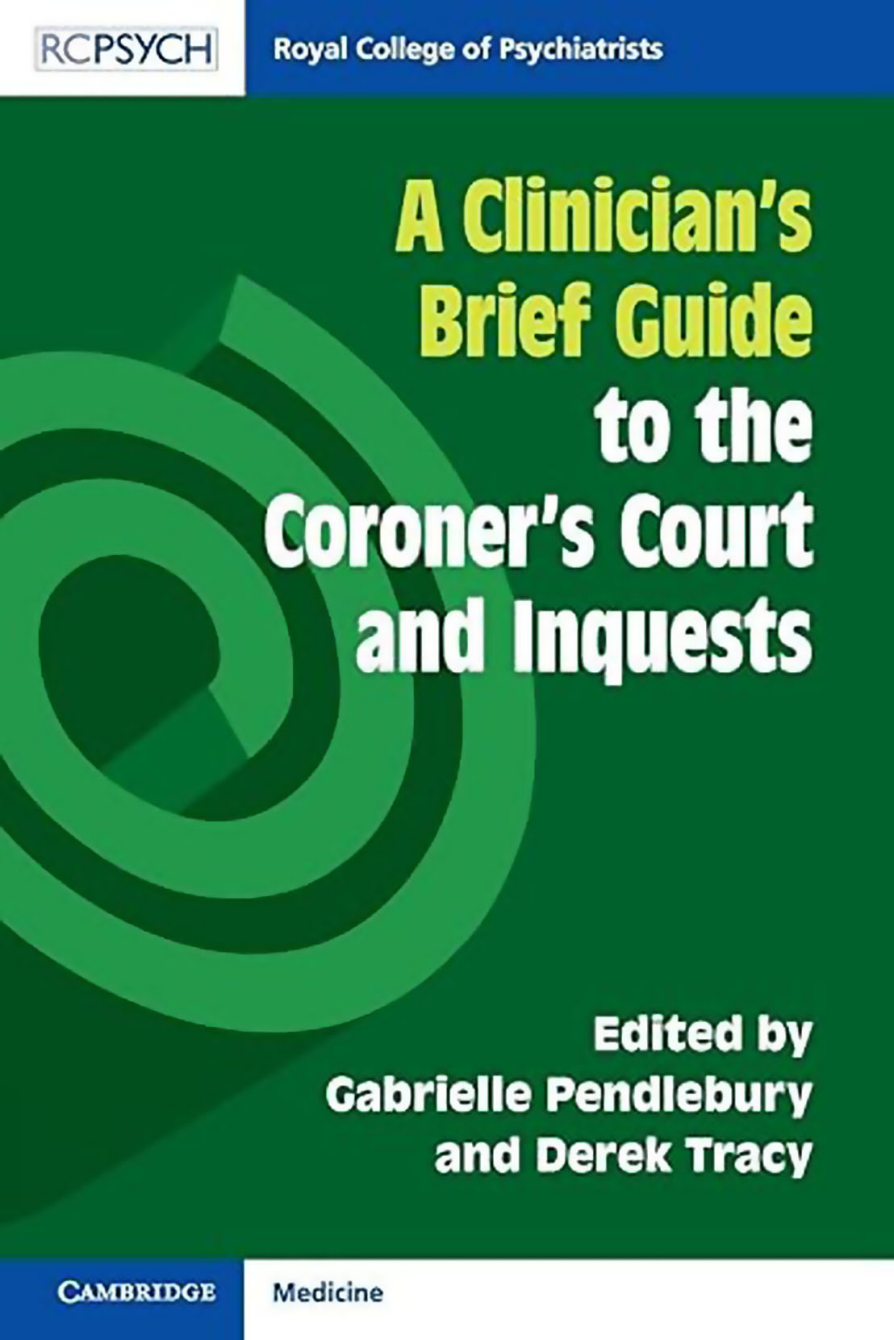

In the past three months I stepped down as president of the Birmingham Medicolegal Society. This role led to my presence at a talk by our local coroner and access to their court for the staging of a mock hearing. I experienced watching the coroner, various lawyers and witnesses interact while taking on the role of a bereaved family member. Couple this with my Forensic Certificate of Completion of Training and I become indisputably biased in favour of books like this. Nevertheless, I argue that it is essential reading for all psychiatrists, and many of the chapters are just as important for all doctors. This is not something I write lightly. I recommended the book to my employing trust’s medical director and resident colleagues when several were called to a particularly emotive hearing as interested persons following a particularly tragic death of a patient in the city where I work.

Every new lobby in our crowded professional world pushes their ideas at our saturated minds. However, given the excess mortality that our patients face and the coronial legal consequences of deaths during detention subject to the Mental Health Act, I don’t think that I am alone in being summoned to give evidence in front of a jury at the coroner’s court as an interested person. Back in 1874 Henry Maudsley wrote that ‘the most anxious cases with which those have to do who are engaged in care and treatment of the insane, are unquestionably those in which there is a persistent suicidal impulse’. The intensity of our doctor–patient relationships coupled with the violence of suicide and homicide can make the coroner’s court an exceptionally daunting place to be called to.

The night before I started working as a foundation doctor in 2005, I feverishly read Bulgakov’s *Notebook of a Country Doctor* from cover to cover. Pendlebury and Tracy’s book perhaps offers similar prophylactic help for all early career doctors or any psychiatrist who doesn’t appear in the coroner’s court regularly. This is due to its brevity, salience and comprehensive coverage of processes. It neatly straddles legalistic precision, day-to-day practicalities and empathic consideration of the psychological impact of the processes. This book is essential reading.

The flow of the book is logical, taking the reader from the notification of death through mandatory NHS patient safety investigations to the actual coroner’s inquest, writing statements, giving oral evidence in the court and outcomes, including adverse outcomes and ways to handle the emotional impact. This is an intuitive and comprehensive journey that you can immerse yourself in prior to in vivo experiences. It involves plenty of high-fidelity technical details, including tough topics, such as when a death is unlawful, when written evidence might not be enough and giving evidence in person will happen, when we should self-refer to the General Medical Council, when we might self-incriminate when giving evidence and who might ask you questions, including jurors. I particularly valued the historical and contextual chapters that help us understand what makes coroner’s courts quite different from other legal settings. The book helpfully touches on unlawful killing, including reference to the experiences of Dr Bawa-Garba, the death of Jack Adock, gross negligence and corporate manslaughter. Perhaps expanding the material on police and criminal investigations into its own chapter might be helpful in future editions.

